# Case Report: Lenvatinib plus osimertinib treated a metastatic hilar cholangiocarcinoma patient harboring EGFR mutation

**DOI:** 10.3389/fimmu.2026.1949186

**Published:** 2026-10-22

**Authors:** Peng Wang, Chentao Zou, Wendi Liu, Jiangli Xu, Hui Wang, Shisan Bao, Huabang Zhou

**Affiliations:** 1 Department of Infectious Diseases, Ruijin Hospital, Shanghai Jiao Tong University School of Medicine, Shanghai, China; 2 Liver Center, Ruijin Hospital, Shanghai Jiao Tong University School of Medicine, Shanghai, China; 3 Department of Hepatobiliary Medicine, Shanghai Eastern Hepatobiliary Surgery Hospital, Shanghai, China

**Keywords:** EGFR mutation, gefitinib, hilar cholangiocarcinoma, lenvatinib resistance, osimertinib

## Abstract

**Background:**

Hilar cholangiocarcinoma (hCCA) is an aggressive malignancy with limited therapeutic options and poor prognosis, particularly in patients with unresectable or metastatic disease. Although immune checkpoint inhibitors and targeted therapies have improved outcomes, resistance remains a major challenge. Here, we report a case of metastatic hCCA with possible lenvatinib resistance managed by sequential molecular-guided therapy.

**Case presentation:**

A 57-year-old man was diagnosed with stage IVB hCCA with extensive distant metastases. As curative surgery was not feasible, gemcitabine plus S-1 chemotherapy was initiated but discontinued after two cycles due to grade 3 myelosuppression. Given high PD-L1 expression (combined positive score = 20), treatment was switched to tislelizumab combined with lenvatinib and S-1, resulting in a partial response of the primary tumor and metastatic lesions. After approximately six months, isolated progression of pulmonary metastases occurred. Retrospective molecular testing of the pretreatment lung biopsy identified an EGFR exon 19 deletion (p.E746_A750del), prompting subsequent EGFR-targeted therapy. Addition of gefitinib achieved marked regression of pulmonary lesions, however, severe drug-related urinary tract inflammation required discontinuation of gefitinib and tislelizumab. Following subsequent progression on lenvatinib monotherapy, treatment was changed to osimertinib plus lenvatinib, resulting in renewed regression of pulmonary metastases. The patient remained on maintenance therapy with sustained disease control and an overall survival exceeding four years.

**Conclusion:**

This case illustrates how molecular reassessment at disease progression can guide subsequent treatment decisions in advanced hCCA. EGFR inhibition may provide clinical benefit in selected patients with lenvatinib-resistant disease, warranting further investigation.

## Introduction

Cholangiocarcinoma (CCA) is anatomically classified into intrahepatic, hilar, and distal subtypes. Among these, hilar cholangiocarcinoma (hCCA), also known as a Klatskin tumour, is the most common subtype, accounting for 40%–60% of all CCA cases (1). hCCA poses considerable therapeutic challenges owing to its propensity to invade critical hilar structures, including major blood vessels, perineural plexuses, lymphatic tissues, and adjacent liver parenchyma. Surgical resection remains the only potentially curative treatment. However, because of the nonspecific nature of its early clinical manifestations, approximately 70% of patients present with locally advanced or metastatic disease at diagnosis, thereby precluding curative surgery (2). Furthermore, even after complete resection, the aggressive biological behaviour of hCCA contributes to high postoperative recurrence rates, with reported 5-year survival rates of less than 40% (3, 4). For patients with unresectable or metastatic disease, gemcitabine plus cisplatin (GC) remains the standard first-line systemic therapy (5, 6). Nevertheless, the prognosis remains poor because of intrinsic and acquired chemoresistance, with a 5-year survival rate below 10%. These findings underscore the urgent need for more effective therapeutic strategies to improve overall survival (OS) in patients with hCCA.

In recent years, molecularly targeted therapies and immunotherapy have shown encouraging clinical efficacy in CCA (7–9). Targeted agents directed against FGFR2 (fibroblast growth factor receptor 2) fusions (7), IDH1**/**2 (isocitrate dehydrogenase 1/2) mutations (8), HER2 (human epidermal growth factor receptor 2) overexpression or amplification (9), BRAF V600E mutations (10), NTRK (neurotrophic receptor tyrosine kinase) fusions (11), and RET (rearranged during transfection) fusions (12) have been approved for biomarker-selected patients. In addition, programmed death-1 (PD-1) or programmed death ligand-1 (PD-L1) inhibitors combined with chemotherapy, with or without lenvatinib, have significantly improved survival outcomes in advanced biliary tract cancer (13–15). However, the clinical applicability of these precision therapies is limited by the relatively low frequency of actionable genomic alterations and their high cost. Although lenvatinib, a multitarget tyrosine kinase inhibitor (TKI) targeting VEGFR1–3, FGFR1–4, PDGFRα, KIT, and RET, has demonstrated promising antitumour activity across multiple malignancies, its clinical efficacy is frequently compromised by the rapid development of acquired resistance. For example, the objective response rate (ORR) of lenvatinib monotherapy is only 24.1% in hepatocellular carcinoma (HCC) (16, 17). Moreover, treatment-related toxicities associated with TKIs and immune checkpoint inhibitors often necessitate dose reduction or treatment interruption, thereby limiting therapeutic efficacy. Consequently, strategies that overcome drug resistance while maintaining acceptable tolerability are essential for improving long-term outcomes in patients with advanced hCCA.

Here, we describe a patient with locally advanced hCCA accompanied by extensive distant metastases who achieved durable disease control and long-term survival despite multiple severe treatment-related adverse events and possible resistance to lenvatinib. Following sequential optimisation of systemic therapy, including EGFR-targeted treatment guided by molecular profiling, the patient experienced sustained tumour remission with good treatment tolerability. This case may highlight a potential therapeutic strategy for improving lenvatinib resistance in advanced hCCA.

## Case presentation

On 10 March 2022, a 57-year-old man was admitted to the Eastern Hepatobiliary Surgery Hospital with progressive jaundice of the skin and sclera, clay-coloured stools, dark tea-coloured urine, and right upper quadrant abdominal pain. He had no history of hepatitis B or C infection or any other significant medical conditions. Physical examination revealed mild jaundice of the skin and sclera without abdominal tenderness, rebound tenderness, palpable abdominal masses, or enlarged supraclavicular lymph nodes.

Laboratory investigations performed on 11 March 2022 demonstrated markedly elevated cholestatic liver enzymes, including gamma-glutamyl transferase (GGT, 3345 U/L; reference range, 10–60 U/L) and alkaline phosphatase (ALP, 1438 U/L; reference range, 45–125 U/L). Serum alanine aminotransferase (ALT, 339 U/L; reference range, 9–50 U/L), aspartate aminotransferase (AST, 206 U/L; reference range, 15–40 U/L), total bilirubin (TBIL, 48.3 μmol/L; reference range, 0–23 μmol/L), and direct bilirubin (DBIL, 42.4 μmol/L; reference range, 0–8 μmol/L) were also elevated. Serum tumour markers, including carbohydrate antigen 19-9 (CA19-9), carcinoembryonic antigen (CEA), and alpha-fetoprotein (AFP), were all within the normal range, as was serum IgG4.

Contrast-enhanced abdominal computed tomography (CT) performed on 12 March 2022 (**Figures 1A–D**) demonstrated irregular thickening of the hilar bile duct wall associated with a periductal soft-tissue mass showing progressive delayed enhancement and marked upstream biliary dilatation. Narrowing of the left portal vein branch and the anterior branch of the right portal vein was also observed. Magnetic resonance cholangiopancreatography (MRCP) (**Figures 1E, F**) revealed complete obstruction of the common hepatic duct and left hepatic duct with abnormal soft-tissue signal intensity at the site of obstruction. Dilatation of the right hepatic duct and intrahepatic bile ducts was also evident. These findings were highly suggestive of hilar cholangiocarcinoma involving the gallbladder neck, with suspected portal lymph node metastasis and invasion of the left and right anterior portal vein branches.

**Figure 1:**
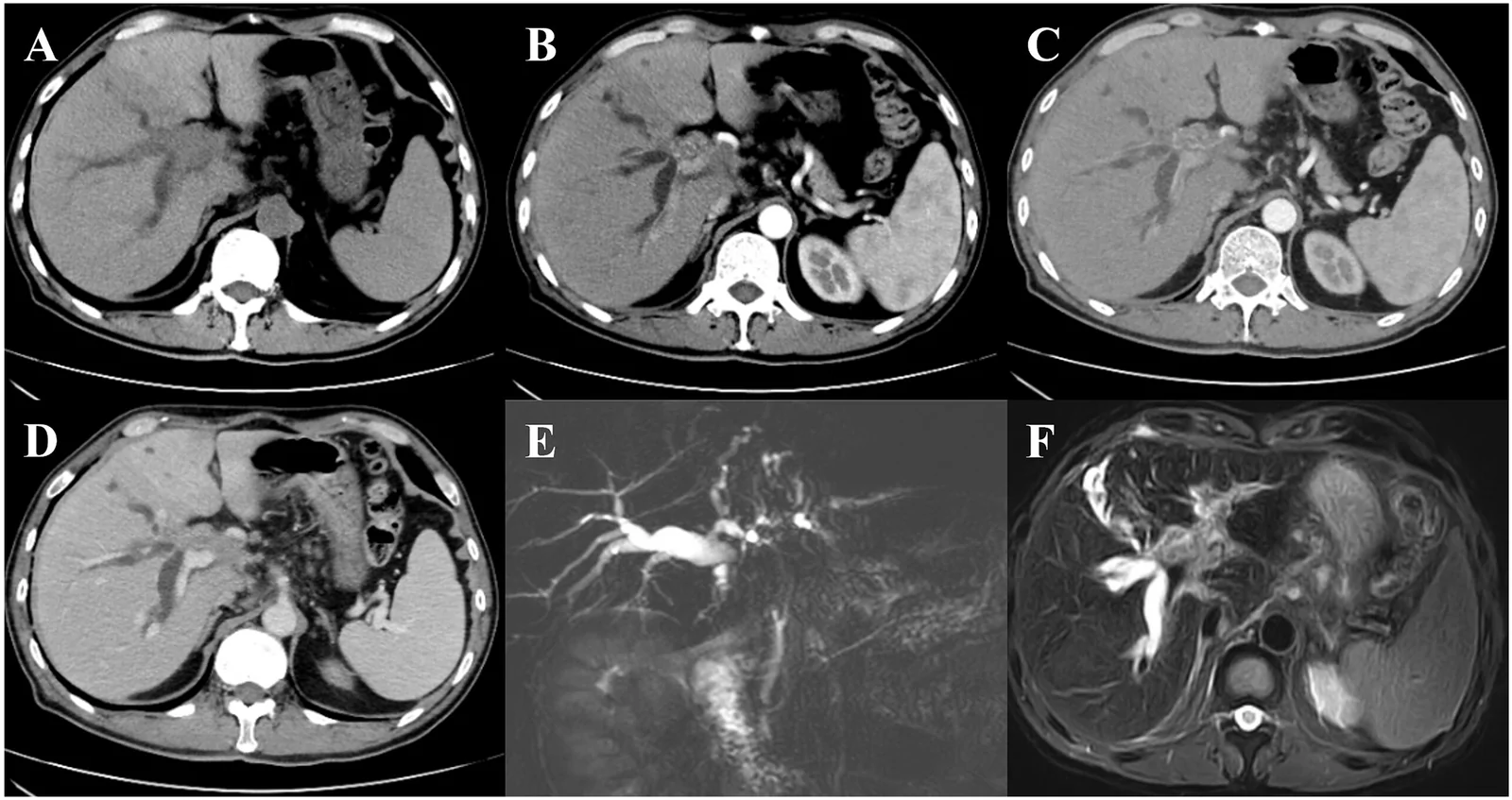
Contrast-enhanced abdominal CT examination, including pre-contrast **(A)**, arterial **(B)**, portal venous **(C)**, and delayed **(D)** phases. MRCP demonstrated marked dilatation of the intrahepatic bile ducts **(E)** and an intraductal soft-tissue lesion in the hepatic hilum **(F)**.

Whole-body positron emission tomography-computed tomography (PET-CT), performed on 14 March 2022 (**Figures 2A–E**), demonstrated a hypermetabolic soft-tissue lesion at the hepatic hilum extending into the intrahepatic bile ducts (SUV_max 6.1), accompanied by narrowing of the hilar bile duct and proximal biliary dilatation. A hypermetabolic pulmonary nodule measuring approximately 3.3 × 2.3 cm was identified in the upper lobe of the right lung (SUV_max 13.8) with irregular spiculated margins. Multiple bilateral pulmonary nodules ranging from 0.2 to 0.6 cm in diameter showed mildly increased FDG uptake (SUV_max 0.9). In addition, multiple enlarged mediastinal and bilateral hilar lymph nodes demonstrated increased metabolic activity (SUV_max 8.1). Multiple osteolytic lesions involving the cervical, thoracic, and lumbar vertebrae as well as the pelvic bones also exhibited markedly increased FDG uptake (SUV_max 8.4). Collectively, these findings suggested hilar cholangiocarcinoma with extensive metastases involving both lungs, mediastinal and hilar lymph nodes, and multiple skeletal sites.

**Figure 2:**
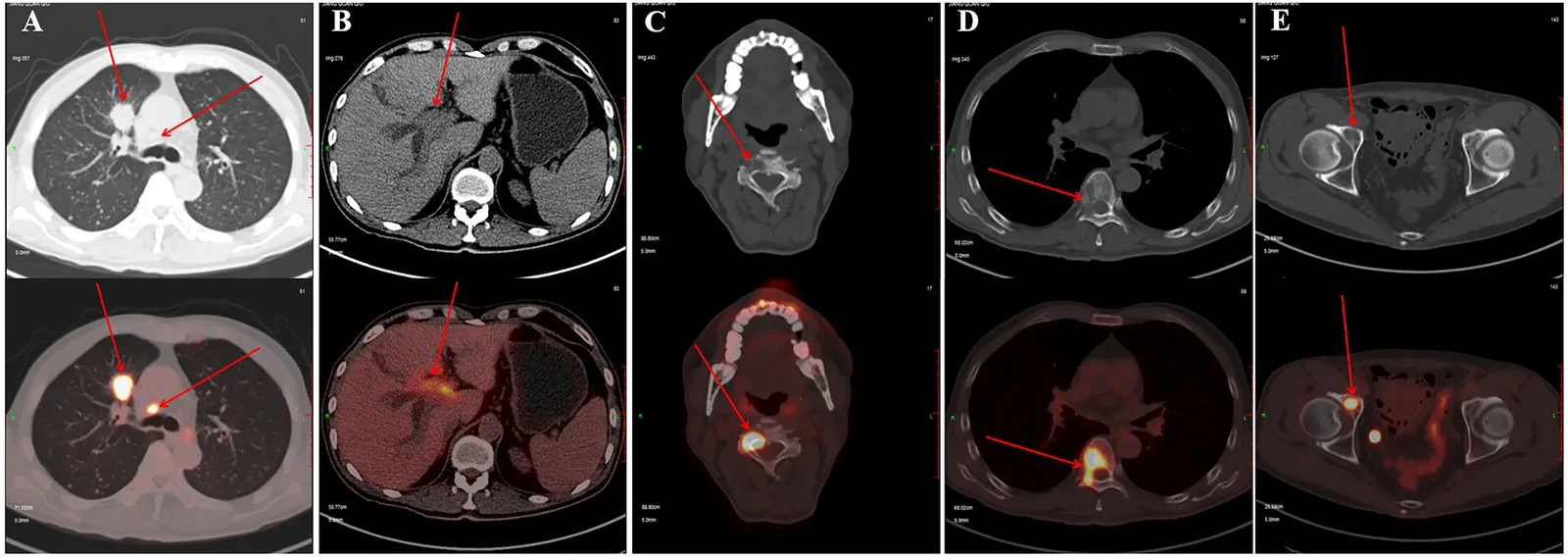
Whole-body PET-CT images. **(A)** A 3.3 cm × 2.3 cm nodular shadow in the right upper lobe with diffuse bilateral small nodular shadows in both lungs and enlarged mediastinal lymph nodes. **(B)** Patchy soft-tissue density at the hepatic hilum. **(C–E)** Multiple patchy osteolytic lesions involving the cervical **(C)**, thoracic **(D)**, and pelvic **(E)** bones.

The patient subsequently underwent endoscopic biliary brushing with metal stent placement and CT-guided biopsy of the right lung lesion. Cytological examination of the biliary brushing identified atypical malignant cells. Histopathological examination of the lung biopsy specimen (**Figures 3A–F**) demonstrated moderately differentiated adenocarcinoma. Immunohistochemical analysis showed that the tumour cells were positive for CK7 (**Figure 3B**), CK19 (**Figure 3C**), MUC1 (**Figure 3D**), CD10, and synaptophysin (Syn), but negative for HepPar-1, CD56, CK5/6, chromogranin A (CgA), ALK, TTF-1 (**Figure 3E**), Napsin A (**Figure 3F**), and p40. PD-L1 expression (antibody clones: E1L3N) showed a combined positive score (CPS) of 20 (**Figure 3G**). Aberrant p53 overexpression was observed in approximately 90% of tumour cells, and the Ki-67 proliferation index was approximately 30%. Based on the clinicopathological and radiological findings, the patient was diagnosed with hCCA, staged as IVB according to the 8th edition of the American Joint Committee on Cancer (AJCC) staging system.

**Figure 3:**
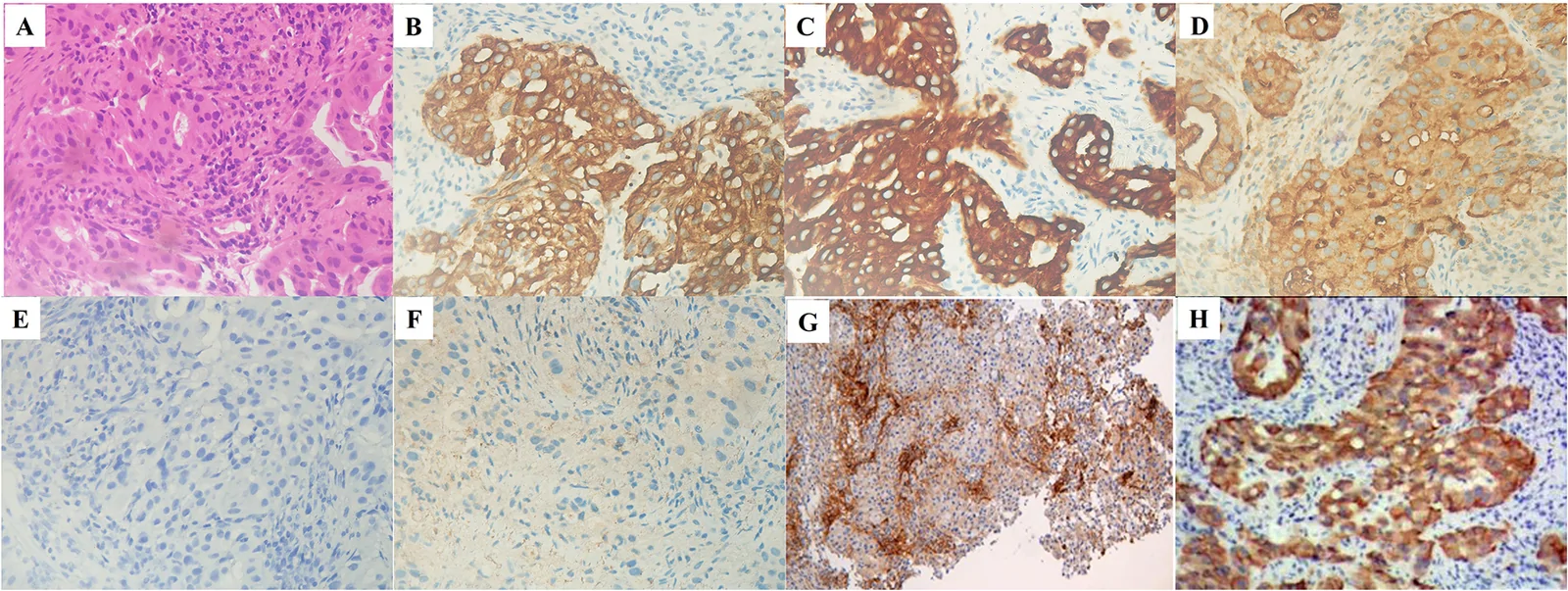
Pathological findings of the lung tumour lesion biopsy. **(A)** Hematoxylin and eosin (H&E) staining (400×) showing moderately differentiated adenocarcinoma (Based on the immunohistochemical results, and in conjunction with the patient’s known biliary tract malignancy, although a primary lung cancer cannot be completely ruled out, it is unlikely). Immunohistochemistry demonstrated strong positive staining for CK7 **(B)**, CK19 **(C)**, MUC-1 **(D)** and negative staining for TTF-1 **(E)** and Napsin A **(F)**. Additional immunohistochemical staining revealed positive PD-L1 expression **(G)** and strong EGFR expression **(H)**.

Because of the locally advanced and metastatic nature of the disease, the patient was considered unsuitable for curative surgical resection. Following multidisciplinary team (MDT) discussion, first-line chemotherapy with gemcitabine plus S-1 (GS) was initiated on 23 March 2022. Gemcitabine was administered intravenously at 1000 mg/m² on days 1 and 8 of each 21-day cycle. S-1 (tegafur/gimeracil/oteracil potassium) was administered orally twice daily on days 1–14, with the daily dose determined according to body surface area (80 mg/day for BSA <1.25 m², 100 mg/day for BSA 1.25–1.49 m², and 120 mg/day for BSA ≥1.50 m²). However, treatment was discontinued after two cycles because of grade 3 myelosuppression (CTCAE 5.0).

Given the high PD-L1 expression, the patient was considered a suitable candidate for immune checkpoint inhibitor therapy. Treatment was therefore switched to a combination of tislelizumab (200 mg, IV infusion, once every 3 weeks), Lenvatinib (8mg orally, once daily), and S-1 on May 16, 2022. After six cycles of treatment, imaging showed marked radiological regression of the primary hilar lesion. Chest CT examinations performed on 7 June 2022 and 16 September 2022 further demonstrated substantial regression of the pulmonary metastatic lesions (**Figures 4A, B**). However, repeat chest CT on 5 December 2022 revealed significant progression of the pulmonary metastases (non-target lesions: PD, overall response: PD) (**Figure 4C**), whereas the primary lesion and bone metastases remained well controlled. Following a second MDT review, based on the NGS results from the patient’s lung puncture tissue specimen dated March 22, 2022—which identified several somatic alterations (3D Medicines, sequencing platform: Illumina NovaSeq 6000, tumor cellularity: 25%; total DNA amount: 861.90 ng; average sequencing depth: 1,519.18×), including an activating EGFR exon 19 deletion (p.E746_A750del, exon 19; variant allele frequency: 12.74%), ARID2 (c.1715 + 1G>C; variant allele frequency: 3.98%), SETD2 (p.Q1911, exon 12; variant allele frequency: 6.32%), and TP53 (p.I232F, exon 7; variant allele frequency: 3.89%), together with a tumor mutational burden (TMB) of 10.06 mutations/Mb—the experts inferred that the patient might be resistant to lenvatinib. Immunohistochemical staining for EGFR was recommended on the previously obtained puncture tissue specimen.

**Figure 4:**
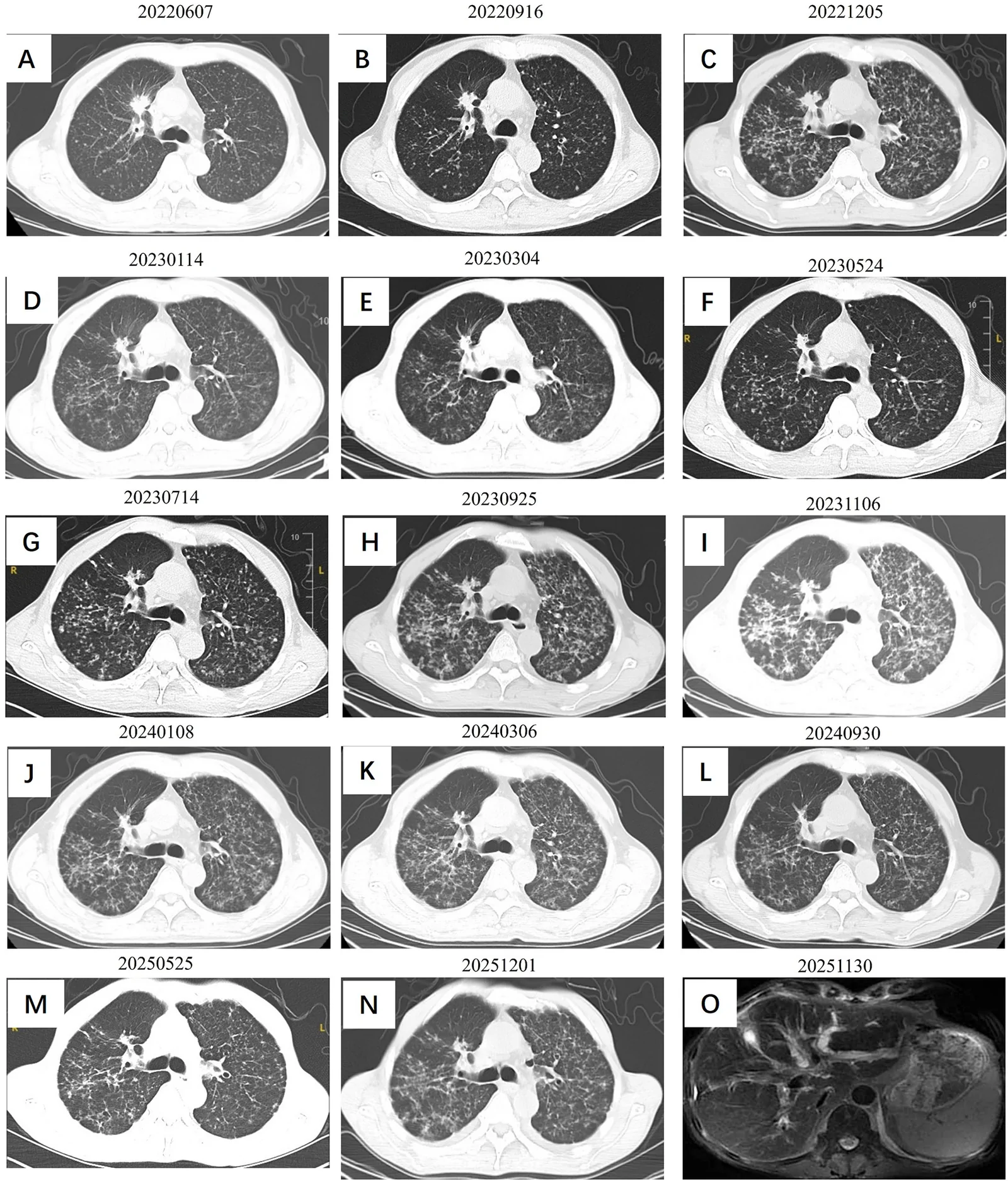
Post-treatment tumor characteristics. **(A–C)** Pulmonary lesion changes with tislelizumab, lenvatinib, and S-1 combination therapy. **(D, E)** Pulmonary lesion changes with tislelizumab, lenvatinib, gefitinib, and S-1. **(F–I)** Pulmonary lesion changes with lenvatinib monotherapy. **(J–N)** Pulmonary lesion changes with lenvatinib plus osimertinib. **(O)** Last follow-up findings of the perihilar cholangiocarcinoma lesion.

Immunohistochemistry demonstrated strong EGFR expression (**Figure 3H**), which prompted the initiation of subsequent EGFR-targeted therapy. After detailed discussion with the patient and his family, treatment was escalated to a four-drug regimen consisting of tislelizumab, lenvatinib, gefitinib (250 mg orally, once daily), and S-1 on December 9, 2022. Following the incorporation of EGFR-targeted therapy into the treatment regimen, pulmonary disease showed a clinical response (non-target lesions: non CR/non PD) (**Figure 4D**). Continued treatment for an additional seven cycles resulted in sustained disease stabilisation (**Figures 4E, F**).

However, since early May 2023, the patient has developed severe flank pain accompanied by gross hematuria, without chills or fever. On May 11, 2023, complete blood count showed WBC 2.94 × 10^9^/L, RBC 3.59 × 10¹²/L, HGB 123 g/L, neutrophil count 1.90 × 10^9^/L, neutrophil percentage 64.7%, and platelets 107 × 10^9^/L; renal function showed blood urea nitrogen 5.42 mmol/L and creatinine 80 µmol/L; urinalysis was light red with urine occult blood 3+, RBC count >5000/µL, and WBC count 2092.1/µL, with no other significant abnormalities. Urine bacterial culture on May 13, 2023, showed no bacterial growth after 2 days of culture, with a urine colony count of 0 CFU/mL. Contrast-enhanced CT urography (CTU) on May 11, 2023, showed bilateral ureteral thickening with surrounding exudation, suggestive of inflammation, and minimal bilateral perinephric exudation. After urological consultation, the most likely diagnosis was suspected treatment-related ureteritis/hematuria, possibly associated with gefitinib or tislelizumab. Consequently, both agents were discontinued (Gefitinib last administered on May 12, 2023; tislelizumab last administered on April 29, 2023) and systemic corticosteroid (Methylprednisolone 80 mg/day) therapy was initiated from May 12, 2023. Three days later, the patient’s right flank pain and gross hematuria had improved markedly, and methylprednisolone was switched to prednisone 10 mg/day. Ureteroscopy performed on May 19, 2023, showed diffuse inflammatory edema and hemorrhage of the ureteral wall, more prominent on the left side. No other significant abnormalities were found in the remaining urinary tract. A 6-Fr double-J stent was therefore placed in the left ureter. The right flank pain resolved after approximately 1 week, and gross haematuria resolved after approximately 1 month. The ureteral stent was removed on July 20, 2023. Six months later, prednisone was adjusted to 5 mg/day and maintained for six months, after which it was discontinued.

Because of the adverse events, the patient subsequently received lenvatinib monotherapy. Unfortunately, follow-up CT performed on July 14, 2023, and September 25, 2023, demonstrated renewed progression of the pulmonary metastases (non-target lesions: PD) (**Figures 4G, H**), with further progression confirmed on November 6, 2023 (**Figure 4I**). PET/CT on September 23, 2023, compared with baseline (March 14, 2022), revealed marked progression of the pulmonary non-target lesions, whereas the pulmonary target lesions showed significantly decreased FDG uptake (SUVmax = 0.5) and a markedly reduced diameter (from 3.3 cm at baseline to 1.3 cm) (**Supplementary Figure A**). FDG uptake in the hepatic hilar lesions was also significantly decreased (SUVmax = 0.7) (**Supplementary Figure B**), and skeletal lesions showed essentially no FDG uptake. (**Supplementary Figures C, D**). A third MDT discussion recommended replacement of gefitinib with the third-generation EGFR tyrosine kinase inhibitor osimertinib while continuing lenvatinib. Treatment was therefore initiated with osimertinib (80 mg orally once daily) plus lenvatinib (8 mg orally once daily) after November 9, 2023.

Follow-up chest CT performed on 8 January 2024 revealed marked radiological regression of the pulmonary lesion (non-target lesions: non CR/non PD) (**Figure 4J**). Serial follow-up CT scans subsequently demonstrated durable disease control of the pulmonary metastases (**Figures 4K–N**), while the primary hilar lesion achieved near-complete radiological remission (**Figure 4O**). At the most recent follow-up in July 2026, the patient remained on maintenance osimertinib and lenvatinib, with sustained disease control and no clinically significant treatment-related adverse events such as pneumonitis or hepatitis. However, after biliary stent placement, three episodes of biliary obstruction and cholangitis occurred, all managed by ERCP with balloon-assisted clearance of the biliary stent. Overall survival exceeded four years.

## Discussion

hCCA, also referred to as perihilar cholangiocarcinoma (pCCA), is characterised by aggressive biological behaviour, complex surgical management, and poor clinical outcomes (6, 18). Complete surgical resection with microscopically negative (R0) margins remains the only potentially curative treatment; however, approximately two-thirds of patients present with unresectable disease at diagnosis because of extensive local invasion or distant metastases (2, 19). In the present case, the patient had stage IVB disease with extensive distant metastases and was therefore unsuitable for curative surgery.

Gemcitabine plus cisplatin (GC) has long been established as the standard first-line systemic therapy for advanced biliary tract cancer following the ABC-02 trial (20). Subsequently, the phase III JCOG1113/FUGA-BT study demonstrated that gemcitabine plus S-1 (GS) achieved non-inferior overall survival compared with GC (15.1 vs. 13.4 months), supporting GS as an alternative first-line regimen, particularly in Asian populations (21). Because this patient was treated in early 2022, before immunochemotherapy had become widely adopted in routine clinical practice, and because GS is associated with a slightly lower incidence of severe adverse events than GC, GS was selected as the initial treatment regimen. However, chemotherapy was discontinued after only two cycles because of grade 3 myelosuppression.

The therapeutic landscape of advanced biliary tract cancer has evolved rapidly in recent years. The addition of immune checkpoint inhibitors to first-line chemotherapy, including durvalumab or pembrolizumab, has significantly improved survival outcomes and has become a new standard of care for advanced disease (13, 14). In parallel, molecularly targeted therapies have expanded treatment options for biomarker-selected patients. Lenvatinib, a multitarget tyrosine kinase inhibitor targeting VEGFR, FGFR, PDGFRα, KIT, and RET, has demonstrated antitumour activity across several solid malignancies and has shown encouraging efficacy when combined with PD-1 blockade in advanced biliary tract cancer (14, 17, 22, 23). Based on the patient’s high PD-L1 expression (CPS = 20), treatment was therefore switched to a combination of tislelizumab, lenvatinib, and S-1. This regimen produced marked radiological regression involving the primary tumour and the pulmonary lesions, together with regression of the bone metastases, supporting the clinical benefit of combined immunotherapy, antiangiogenic therapy and chemotherapy.

Despite this favourable initial response, pulmonary metastases progressed after approximately six months of treatment, whereas the primary hilar lesion and osseous metastases remained well controlled. This discordant response likely reflects the marked spatial and biological heterogeneity of advanced hCCA and highlights the emergence of resistance during systemic therapy. Resistance to lenvatinib has increasingly been recognised as an important limitation of long-term treatment efficacy. Although the underlying mechanisms remain incompletely understood, activation of compensatory signalling pathways, particularly EGFR signalling, has been implicated in preclinical studies as a potential mechanism of resistance (16, 24–27).

To investigate the mechanism underlying disease progression during treatment with tislelizumab, lenvatinib, and S-1. Retrospective molecular testing of the pretreatment lung biopsy identified an EGFR exon 19 deletion (p.E746_A750del). Although activating EGFR mutations are uncommon in cholangiocarcinoma, their presence suggested that EGFR signalling might have contributed to the continued progression of the pulmonary metastases. Based on EGFR mutations, prompting subsequent EGFR-targeted therapy, gefitinib was added to the treatment regimen. The patient experienced rapid symptomatic improvement, accompanied by marked radiological regression of the pulmonary lesions, whereas disease control at the primary tumour and bone metastases was maintained. Although this favourable response cannot establish a causal relationship between EGFR activation and lenvatinib resistance, it is consistent with previous experimental studies suggesting that EGFR pathway activation may represent a bypass mechanism that attenuates the antitumour activity of lenvatinib (16, 25–27).

Unfortunately, treatment was complicated by severe drug-related urinary tract inflammation manifested by flank pain and gross haematuria, necessitating discontinuation of gefitinib and tislelizumab. Disease progression recurred shortly after treatment de-escalation despite continuation of lenvatinib monotherapy, further supporting the importance of EGFR inhibition in controlling the pulmonary metastatic lesions. Considering the favourable initial response to EGFR-targeted therapy and the superior efficacy and safety profile of third-generation EGFR tyrosine kinase inhibitors, gefitinib was subsequently replaced with osimertinib. Osimertinib irreversibly inhibits both classical EGFR-sensitising mutations and the T790M resistance mutation and has demonstrated superior efficacy, improved central nervous system penetration, and better tolerability compared with first-generation EGFR inhibitors in non-small cell lung cancer (28–30). Following initiation of osimertinib plus lenvatinib, the patient again achieved sustained regression of the pulmonary metastases without recurrence of severe treatment-related adverse events. More importantly, durable disease control has been maintained for more than four years from the initial diagnosis.

To our knowledge, reports describing the successful use of osimertinib to overcome suspected lenvatinib resistance in advanced hCCA remain extremely limited. Although the underlying mechanism requires further validation, the clinical response suggests that EGFR-directed therapy may provide benefit in selected patients with lenvatinib resistance, it illustrates how comprehensive molecular profiling performed at the time of disease progression may identify actionable alterations that facilitate rational treatment optimisation. This case also highlights the potential value of sequential precision medicine strategies for patients with advanced biliary tract cancer who develop resistance to systemic therapy.

Admittedly, this case report has two important limitations. First, we could not obtain tissue specimens from the hCCA and therefore could not compare them with the pulmonary tumour lesions. The possibility that the pulmonary lesions originated from cholangiocarcinoma was inferred solely from immunohistochemical findings; however, primary lung cancer could not be completely excluded. Second, the suspicion of lenvatinib resistance was based on pretreatment testing of pulmonary tumour tissue. On the one hand, this could not distinguish whether the progression of non-target lesions was due to primary or acquired resistance to lenvatinib. On the other hand, because gefitinib, as part of the combination therapy, re-induced remission of the pulmonary metastases, it remains unclear whether gefitinib acted synergistically with other agents or genuinely reversed lenvatinib resistance. These questions warrant further clinical observation and mechanistic investigation.

## Data Availability

The original contributions presented in the study are included in the article/**Supplementary Material**. Further inquiries can be directed to the corresponding authors.
