# Supplementary material for: Case Report: Lenvatinib plus osimertinib treated a metastatic hilar cholangiocarcinoma patient harboring EGFR mutation
Source: Front Immunol. 2026 Oct 22;17:1949186. doi: 10.3389/fimmu.2026.1949186 (PMC13735606; doi:10.3389/fimmu.2026.1949186)
Supplement: Supplementary file 1 [file DataSheet1.pdf]

Supplementary figure

PET-CT Changes in Tumor Lesions on September 23, 2023, Compared with the Pretreatment Baseline (March 14, 2022). PET/CT on September 23, 2023, compared with baseline (March 14, 2022), revealed marked progression of the pulmonary non-target lesions, whereas the pulmonary target lesions showed significantly decreased FDG uptake (SUVmax = 0.5) and a markedly reduced diameter (from 3.3 cm at baseline to 1.3 cm) (A). FDG uptake in the hepatic hilar lesions was also significantly decreased (SUVmax = 0.7) (B), and skeletal lesions showed essentially no FDG uptake (C and D).
